# DNA priming immunization is more effective than recombinant protein vaccine in eliciting antigen-specific B cell responses

**DOI:** 10.1080/22221751.2021.1918026

**Published:** 2021-05-01

**Authors:** Haiying Li, Shixia Wang, Guangnan Hu, Lu Zhang, Shuying Liu, Shan Lu

**Affiliations:** a Laboratory of Nucleic Acid Vaccines, Department of Medicine, University of Massachusetts Medical School, Worcester, MA, USA; b Department of General Surgery, The First Affiliated Hospital, Nanjing Medical University, Nanjing, Jiangsu, People’s Republic of China; c SYL-Consulting, Thousand Oaks, CA, USA

**Keywords:** HIV-1, envelope glycoprotein, DNA vaccine, protein vaccine, heterologous prime – boost, antibody, B cell

## Abstract

While DNA prime-protein boost vaccination approach has been widely used in preclinical and clinical studies especially in the field of HIV vaccine development, the exact role of DNA immunization has not been fully identified. Our previous work demonstrated that DNA immunization was able to elicit T follicular helper (Tfh) cell responses and germinal center (GC) B cell development in a mouse model. In the current report, a mouse immunogenicity study was conducted to further ask whether DNA immunization is able to elicit antigen-specific B cell responses. Using HIV-1 Env as model antigen delivered in the form of DNA prime-protein boost, our data demonstrated that DNA prime was able to enhance the antigen-specific B cell responses for both Env-specific antibody secreting cells (ASC) and memory B cells. Furthermore, the DNA priming can greatly reduce the need of including an adjuvant as part of the recombinant protein vaccine boost formulation. Our findings revealed one mechanism that supports the value of DNA priming in assisting the inductin of high affinity and long lasting antigen specific antibody responses.

## Introduction

Inducing robust and long-lasting antibody response is the major objective as well as the challenge in HIV vaccine development. In our previous preclinical and clinical studies, the DNA prime-protein boost approach was shown to be effective in eliciting high levels and better qualities of HIV-1 specific antibody responses which were fairly long lasting within the study period [1–5]. In order to understand the roles of DNA immunization in such an approach, we further demonstrated in a mouse model that HIV-1 envelope glycoprotein (Env) DNA vaccine was more effective than the recombinant Env protein vaccines in eliciting germinal center B cell responses [6,7]. Along with such observation, Env DNA prime followed with Env protein boost was able to elicit higher levels of antibody responses than Env protein alone vaccination [6,7], and DNA priming was able to increase the antibody avidity in a separate small animal study [5]. However, in those studies, we did not investigate to what degree the Env specific B cells were activated by DNA immunization. Finding the answer to this question may hold the key to better understand the mechanism of DNA vaccination.

In the current study using the mouse model, the levels of gp120-specific antibody-secreting plasma cells (ASC) and memory B cells elicited by the gp120 DNA prime–gp120 protein boost approach were evaluated relative to either DNA alone or protein alone immunizations. Because activation and development of antigen-specific B cells are critical for the high affinity and long-lasting antibody responses, our study would shed light on the mechanism of DNA immunization to the development of high quality antibody responses for HIV vaccines. In addition, in animals primed with the gp120 DNA vaccine, we also studied whether the protein boost can be equally effective in eliciting high level antibody responses in the absence of an adjuvant. Our aim is to establish the unique contribution of DNA immunization in the development of antigen-specific B cells and ultimately the development of high titer and high quality antibody responses.

## Materials and methods

### gp120 DNA vaccine

The plasmid DNA vaccine encoding the consensus HIV-1 gp120 antigen from subtype BC (gp120-BC) was constructed utilizing the DNA vaccine vector pJW4303 under the human tissue plasminogen activator (tPA) leader [8]. The expression of gp120-BC protein by DNA vaccine was verified by transient transfection of 293T cells and Western blot analysis as previously reported [2]. The gp120-BC DNA vaccine was purified using QIAGEN Mega DNA prep kit (Valencia, CA) for mouse immunization studies.

### Recombinant gp120 protein vaccine

The recombinant gp120-BC protein was produced by transient transfection of FreeStyle™ 293F suspension cells (Invitrogen, Carlsbad, CA) [9] using the same gp120-encoding DNA vaccine plasmid. In brief, cells were transfected at a density of 1 × 10^6^/ml in GIBCO® FreeStyle™ 293 expression media using 293fectin™, according to manufacturer’s instructions (Invitrogen). Three days after the transfection, supernatant was collected and the gp120 protein was purified by lentil-lectin affinity chromatography (GE Healthcare, Chicago, IL). The purified gp120-BC protein was verified by ELISA and Western-blot analysis.

### Mouse DNA and protein immunization

**Figure 1.**
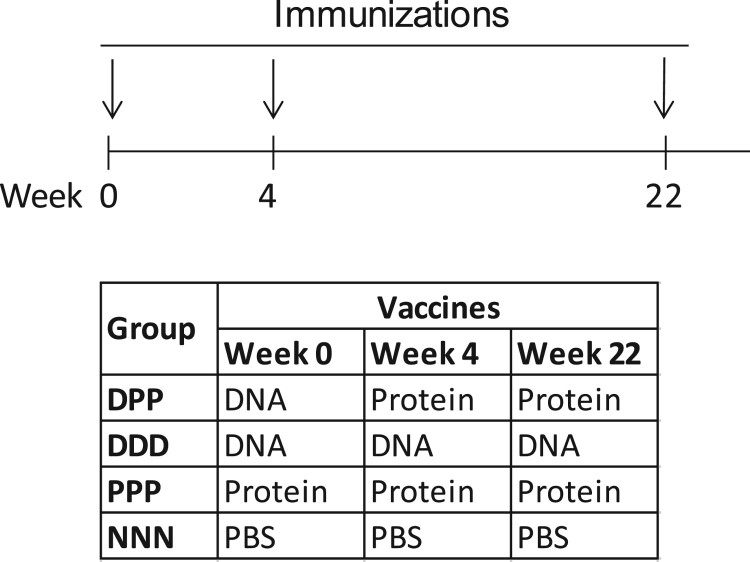
Study design for testing gp120-specific B cell responses with different immunization regimes. Four groups of mice (N* *= 15 per group) received intramuscular injection at weeks 0, 4 and 22 as indicated. DPP: gp120 DNA prime-protein boost; DDD: DNA alone; PPP: protein alone; NNN: PBS control.

Six to eight week old C57/BL6 mice (5–15 mice/group) were purchased from Taconic Farms (Germantown, NY) and housed in the Department of Animal Medicine at the University of Massachusetts Medical School (UMMS) in accordance with IACUC approved protocols. The immunization schedules for different studies are shown in Figures 1, 4(A) and 6, respectively. Both DNA and protein vaccines were administered by conventional intramuscular needle injection. For DNA immunization, each mouse received 200 µg of gp120-BC DNA in saline at each time point. For protein immunization, each mouse received 5 µg recombinant gp120-BC protein at each time point. Adjuvant aluminium hydroxide (Alum) (Sigma, St. Louis, MO) with a dose of 50 μg/mouse was formulated with gp120-BC protein in most mouse groups (except Group Dpp-8) shown in Figures 1, 4(A) and 6. The negative control animals in Group NNN (Figure 1) and Group NNN-8 (Figure 5) received 100 μl PBS at each immunization and the Alum control group (AAA) received 50 μg Alum at each immunization. Serum samples were collected prior to the start of study and at peak time (1 week after the last immunization) for gp120-specific antibody analysis. The bone marrow cells and splenocytes were collected at ∼ 2 weeks after the last immunization to measure the antigen-specific B cell responses.

### Enzyme-linked immunosorbent assay (ELISA)

ELISA was performed to measure the gp120-BC specific IgG antibody responses in mouse sera. The 96-well microtiter plates were coated with 100 μl of gp120-BC protein at 1 μg/ml in PBS (pH 7.2) for 1 hour at room temperature (RT), and then blocked with 5% milk/4% whey blocking buffer at 4°C overnight. Serially diluted mouse sera (100 μl/well) were added to the plates in duplicate and incubated at RT for 1 hour. Subsequently, the plates were incubated with 100 μl/well of biotinylated anti-mouse IgG (Vector Labs, Burlingame, CA) at 1:1000 at RT for 1 hour; followed by incubation with 100 μl/well of horseradish peroxidase (HRP)-conjugated streptavidin (Vector Labs) at 1:2000 in dilution buffer at RT for 1 hour. All samples were diluted in 4% whey dilution buffer and 5 times of washes were applied between steps using washing buffer (0.1% Triton-X in PBS). Finally, the plate was developed with 3,3’,5,5’-Tetramethybenzidine (TMB) solution for 3.5 minutes and stopped with 25 μl of 2 M H_2_SO_4_. The plates were read by ELISA reader at OD450 nm. The gp120-BC specific antibody titers were determined as the reciprocal dilution of serum with OD values two times greater than the pre-immune serum sample. The IgG isotype-specific ELISA for IgG, IgG1, IgG2b and IgG2c were performed, as previously described [10]. This assay is similar to the above, with the exception that HRP-conjugated goat-anti-mouse IgG, IgG1, IgG2b or IgG2c (SouthernBiotech, Birmingham, AL) at 1:2000 dilution was used. The concentrations for gp120-specific mouse IgG, IgG1, IgG2b, or IgG2c were calculated based on the standard curves using a known amount of purified mouse IgG, IgG1, IgG2b or IgG2c (Southern Biotech), respectively.

### B cell ELISPOT

ELISPOT was performed to measure the number of either total IgG-secreting, or gp120-specific antibody secreting cells (ASC) in splenocytes or bone marrow cells as previously described [11–13]. Briefly, 96-well MultiScreen-IP filter plates (Millipore, Burlington, MA) were coated with 100 μl of gp120 protein at 30 μg/ml in PBS or goat anti-mouse Ig at 5 μg/ml in PBS (Mabtech, Cincinnati, OH), at 4°C overnight. Plates were then blocked with RPMI/10% FBS (R-10) media at 37°C for 2 h. The mouse splenocytes or bone marrow cells (2×10^5^ cells/well in 100 μl of R-10 media) were added to the plates, and incubated for 16 h at 37°C/5%-CO_2._ Subsequently, the plates were incubated with a biotinylated anti-mouse IgG antibody (Mabtech) at 1:1000 dilution in 2% FBS for 2 h at room temperature and followed by incubation with HRP-conjugated Avidin-D (Vector Labs) at 1:2000 dilution in 2% FBS. The plates were developed with AEC substrate (3-amino-9-ethylcarbazole; Sigma-Aldrich). The sample volume was 100 μl/well in all steps and the plates were washed 3 times with PBS (200 μl/well) between steps. After the developed plates dried in the dark, the plates were scanned and analysed using an automated ELISPOT counter (Cellular Technologies, Ltd., Shaker Heights, OH). The number of spots per million cells was calculated.

To detect the memory B cells as previously described [11,12], the bone marrow cells or splenocytes were first stimulated for 5 days. Briefly, the bone marrow cells or splenocyte cells were cultured in 24-well plates at 1×10^6^ cells/well in 1 ml of R-10 media supplemented with an optimized mix of polyclonal mitogens including 1 μg/ml R848 (Mabtech) and 10 ng/ml recombinant human IL-2 (Mabtech). R-10 media only without stimulation was used as negative controls. After 5 days of culture, the cells were harvested and washed with R-10 media before adding to the ELISPOT plate as described in the above ELISPOT assay.

### Statistical analyses

One-way ANOVA was used to compare the average natural log-transformed gp120-specific antibody titers, IgG concentrations and number of B cells among groups. The *p* value less than 0.05 (*p <* 0.05) was considered as a significant difference.

## Results

### DNA prime-protein boost immunization elicited high levels of gp120-specific B cell responses in addition to serum anti-gp120 antibody responses

In order to understand the impact of vaccination approach to the development of antigen-specific B cell responses, the first part of the study included four groups of mice, each receiving one of the following immunization designs: one DNA plus two protein immunizations (DPP), three DNA immunizations (DDD), three protein immunizations (PPP), or the negative control with three times PBS injections (NNN). All groups followed the same delivery schedule at Weeks 0, 4 and 22 (Figure 1). The gp120 glycoprotein of HIV-1 CRF07_BC consensus, in the form of DNA and protein vaccines, was used as the model immunogen in the current study. Traditionally an adjuvant is included in recombinant protein-based subunit vaccines while live attenuated vaccines and most inactivated vaccines do not require the use of an adjuvant. Therefore, an adjuvant has also been included as part of the protein boost as in our previous DNA prime-protein boost immunization studies [1–3,5]. In the current study, Alum was selected as the adjuvant for the protein boosts because it is widely used as part of multiple recombinant protein vaccine formulations licensed for human clinical applications such as hepatitis B virus vaccine (RECOMBIVAX HB^®^) and human papilloma virus vaccine (GARDASIL^®^) [14,15].

**Figure 2.**
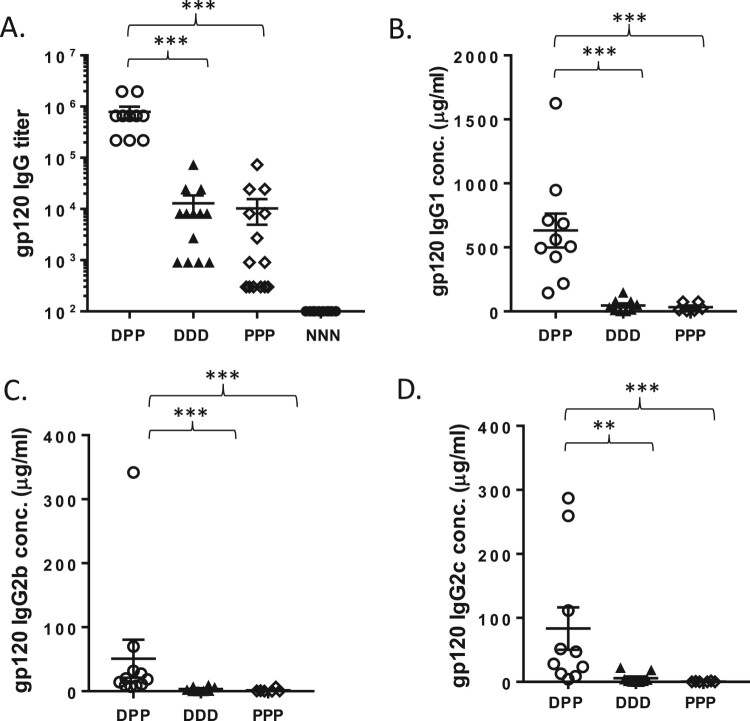
Mouse serum gp120-specific antibody responses. The gp120-specific IgG titer (A), gp120-specific IgG1 concentration (B), gp120-specific IgG2b concentration (C) and gp120-specific IgG2c concentration (D) were measured by ELISA. The statistical significance between different vaccination regimens is indicated, ** as *p <* 0.01 and *** as *p <* 0.001.

Peak level serum antibody responses were measured at Week 23, 7 days after the last immunization. The DNA prime-protein boost group (DPP) elicited the highest level of gp120-specific antibody responses, with the group average titer of 1:656,000 (Figure 2(A)), about 10-fold higher than either the DNA alone or protein alone group (*p*<0.001 compared to either group). The DNA prime-protein boost group (DPP) also elicited significantly higher levels of serum IgG1, IgG2b and IgG2c responses than those in DNA alone group (DDD) or protein alone group (PPP) (Figure 2(B–D)).

**Figure 3.**
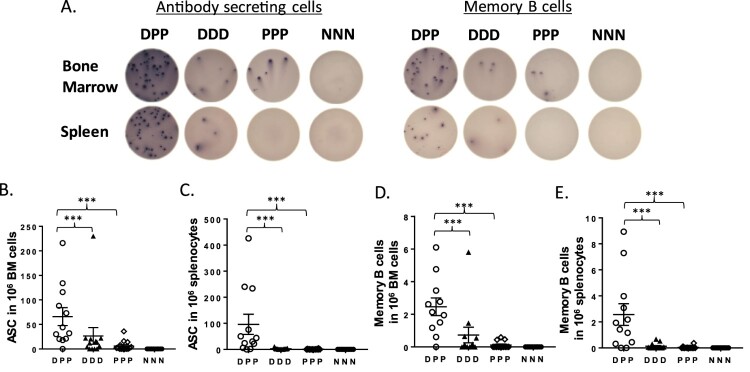
The gp120-specific B cell responses as measured by B cell ELISPOT, including both antibody secreting cells (ASC) and memory B cells in bone marrow and spleen compartments. (A) Representative B-cell ELISPOT readouts from each immunization group; (B) ASC in bone marrow (BM); (C) ASC in spleen; (D) memory B cells in bone marrow; and (E) memory B cells in spleen. The statistical significance between different vaccination regimens is indicated, *** as *p <* 0.001.

There is a critical need to understand how antigen-specific B cells develop into antibody-secreting plasma cells (ASC) and long-lived memory B cells induced by different vaccination approaches. In the current study, we used B cell ELISPOT assay to assess ASC responses. We also measured the memory B cell responses by first stimulating mouse B cells with R848 and IL-2 in vitro to achieve B cell expansion and differentiation into ASC prior to adding the B cells into the ELISPOT plate. Both spleen and bone marrow samples were used for ASC and memory B cell analysis (Figure 3(A)). The DNA prime-protein boost group (DPP) had significantly higher levels of gp120-specific ASC (Figure 3(B,C)) and memory B cell (Figure 3(D,E)) responses than the DNA alone (DDD) or protein alone (PPP) approaches. With in vitro stimulation and culture for 5 days, the total numbers of surviving B cells (memory B cells) for all groups were lower than the fresh B cell analysis (ASC), but the relative difference between DPP and DDD/PPP is similar for both ASC and memory B cell populations.

### The impact of time interval during prime and boost immunizations to elicit the high gp120-specific antibody responses by DNA prime-protein boost strategy

**Figure 4.**
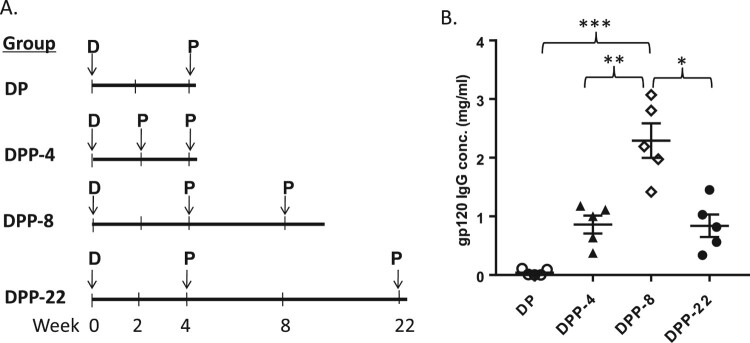
(A) Immununization designs to study the impact of vaccination intervals for various prime-protein boost regimens (N* *= 5 per group). (B) Mouse serum gp120-specific antibody concentration was measured by ELISA. The statistical significance between different vaccination regimens is indicated, * as *p <* 0.05, ** as *p <* 0.01 and *** as *p <* 0.001, respectively.

In the above study, we adopted an immunization schedule at Weeks 0, 4 and 22, following the design for conventional subunit vaccines such as HBV and HPV vaccines with the first two immunizations delivered close to each other and the third immunization after a long resting period. For such traditional vaccines, the prime and boost are homologous as the same types of vaccines are used. We next examined what is the optimal resting period for the heterologous DNA prime-protein boost approach. C57BL/6 mice were immunized with the gp120-BC DNA prime-protein boost vaccine as described above but the inoculation time frame varies (Figure 4(A)). In addition to DDP-22 group as the base line immunization schedule as in the above study (DNA once at Week 0 and protein twice at Weeks 4 and 22), DP group received only two immunizations (DNA at Week 0 and protein at Week 4) without the final boost. DPP-4 received two closely placed protein boosts at Weeks 2 and 4 after DNA immunization at Week 0. DPP-8 delivered last protein boost at Week 8 instead of Week 22 comparing to the baseline group (Figure 4(A)).

Serum was collected at 7 days after the last immunization for all study groups and the gp120-specific IgG levels were examined. Not surprisingly, a total of two immunizations including one protein boost induced a lower level of gp120-specific antibody response than other groups with two protein boosts (Figure 4(B)). Among groups with three immunizations, the timing of boosts appears to be critical. DPP-8 elicited a significantly higher level of gp120-specific antibody responses than either DPP-22 with the late last boost or DPP-4 with closely clustered protein boosts (*p *= 0.016 and *p *= 0.009, respectively).

### The role of adjuvant in a DNA prime-protein boost approach

**Figure 5.**
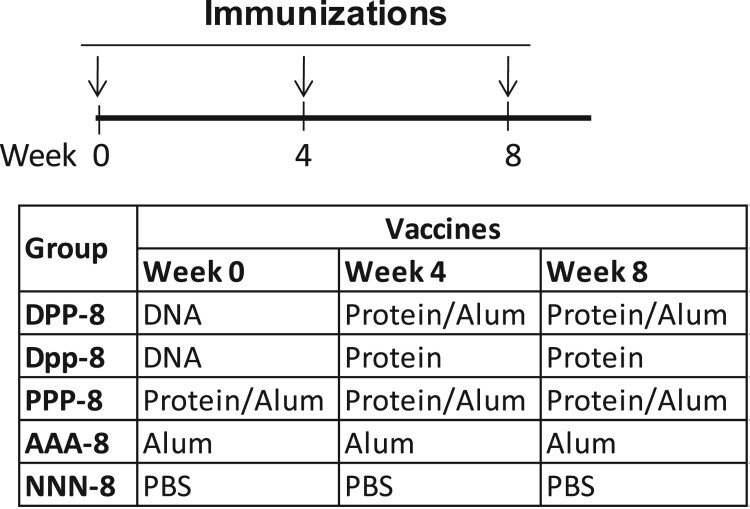
Study design to test gp120-specific B cell responses elicited by DNA prime-protein boost with (DPP-8) or without (Dpp-8) the Alum adjuvant. Protein alone with Alum (PPP-8), Alum only (AAA-8) and PBS (NNN-8) were used as controls (N* *= 5 per group).

**Figure 6.**
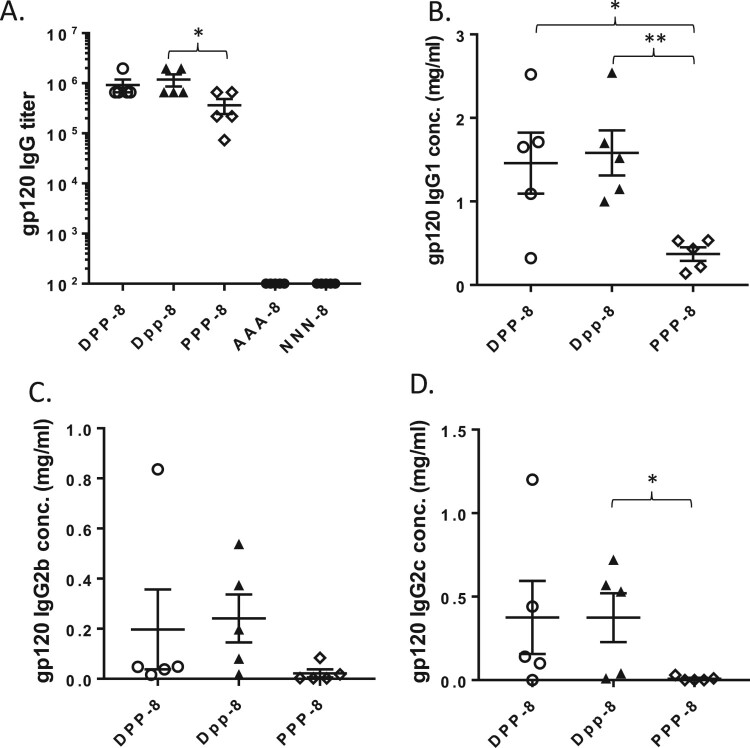
Mouse serum gp120-specific antibody responses induced by different immunization regimens. The gp120-specific IgG titer (A), gp120-specific concentrations of IgG1 (B), IgG2b (C) and IgG2c (D) were measured by ELISA. The statistical significance between different vaccination regimens is indicated, * as *p <* 0.05 and ** as *p <* 0.01, respectively.

We then explored the role of adjuvant in the DNA prime-protein boost approach. C57BL/6 mice were immunized with DNA prime-protein boost using the Weeks 0-4-8 schedule either with alum (DPP-8) or without alum (Dpp-8) (Figure 5). Animals receiving protein vaccine alone (PPP-8), alum alone (AAA-8) or saline (NNN-8) were included as the controls. Mouse immune sera were collected at 7 days after the last immunization and gp120-specific IgG responses were measured. As expected, DNA prime-protein boost with alum (DPP-8) was highly immunogenic as reflected by the high titer gp120-specific IgG responses (Figure 6(A)). The protein alone immunization with alum (PPP-8) also raised high titer gp120-specific IgG response but lower than the DPP-8 group (Figure 6(A)). But it was quite surprising to observe that the levels of gp120-specific IgG responses elicited by the DNA prime-protein boost without adjuvant alum (Dpp-8) were also very similar to the group with adjuvant (DPP-8) (Figure 6(A)). Analysis of IgG subclasses also showed that DPP-8 and Dpp-8 induced similar levels of gp120-specific antibody responses and both were more effective than the protein alone group (PPP-8) (Figure 6(B–D)).

**Figure 7.**
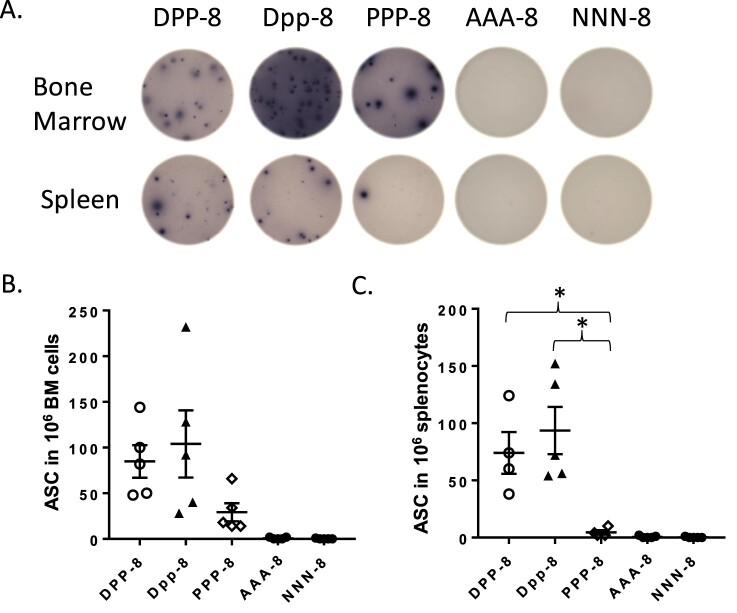
The gp120-specific B cell responses as measured by B cell ELISPOT. (A) Representative readouts for ASC in bone marrow and spleen from different immunization groups. Quantitative ASC comparison among different groups for spleen (C) and bone marrow (BM) samples (B). The statistical significance between different vaccination regimens is indicated, * as *p <* 0.05.

Further analysis with B cell ELISPOT confirmed that the DNA prime-protein boost approach with Alum (DPP-8) or without Alum (Dpp-8) regimens were equally effective in eliciting higher gp120-specific B cells than protein alone with Alum group (PPP-8) (Figure 7(A)). The same pattern of difference was observed for gp120-specific ASC response from either spleen (Figure 7(C)) or bone marrow (Figure 7(B)) sourced B cells but statistical significance was only found with spleen B cells but not bone marrow B cells.

## Discussion

Developing an effective HIV-1 vaccine remains to be a major scientific challenge even after several decades’ effort. The traditional live attenuated and inactivated vaccines were considered either not safe or not sufficiently immunogenic against conformational antigens. The failure of STEP trial had led many scientists to question the feasibility of an HIV vaccine mainly based on T-cell responses, especially when such responses were elicited by viral vector based vaccines such as Ad5 vector [16,17]. RV144 trial, the first partially protective HIV vaccine, renewed the hope for vaccines based on antibody mediated protection [18]. However, protection in RV144 waned rapidly by the end of the 2–3 years follow-up [19–21], which raised major concern on how to elicit robust and long-lasting antibody response by HIV vaccines. The recent failure of HVTN702 to elicit protection while trying to reproduce the results of RV144 further validated such concern [22].

Significant efforts in HIV vaccine development have focused on the design of structure-based Env antigens based on the study of broadly neutralizing monoclonal antibodies. Such effort is valuable and has added much to our understanding on how to elicit antibody responses against particular antigen epitopes by vaccination. However, limited effort has been devoted to the overall improvement on the general quality of antibody responses such as antibody affinity and longevity of antibody responses by candidate HIV vaccines. More importantly, work is needed to identify optimized approaches to elicit antigen-specific B cell responses which is critical for efficient antibody maturation and improved antigen-specific memory B cell development. Our previous mouse studies demonstrated that the DNA immunization was effective in enhancing germinal center B cell reaction, presumably influenced by the elevated Tfh cell responses observed in the same study [6,7], and DNA immunization was able to induce high avidity Env-specific serum antibody responses in a rabbit model [5]. Our pilot human study with the DNA prime-protein boost HIV vaccine (DP6-001) was effective in eliciting a high magnitude of antibody response in 100% of clinical trial volunteers and the levels of antibodies were persistent for at least 6 months after the last vaccination [3]. However, it was not known whether DNA immunization can directly affect the levels of Env-specific B cell responses.

In the current study, by using the envelope glycoprotein gp120 of HIV-1 CRF07_BC consensus as a model antigen, we analyzed the levels of vaccine-induced Env-specific ASCs and memory B cells along with Env-specific antibody responses in C57BL/6 mice after immunization with different DNA and protein vaccine designs. Several important new findings are observed.

First, DNA priming plays a key role in stimulating antigen-specific B cells development including both ASC and memory B cells. This finding is highly important because it answers a key question about the real value of DNA vaccines which are not immunogenic enough when used alone in human clinical studies but highly effective in priming the human immune responses when used in combination with another vaccine modality such as recombinant protein vaccines [3,23–31]. It suggested that DNA immunization prepares high levels of antigen-specific B cells which are the basis for a high magnitude antibody response. This finding is consistent with our previous reports that DNA vaccine is capable of eliciting high levels of Tfh cells and germinal center B cells [6,7] and DNA vaccine can also use innate immunity pathways to activate antigen specific immune responses [32,33]. Our data are also consistent with previous reports that DNA immunization was able to elicit gp120 antibody responses with high avidity which requires more advanced maturation of antigen-specific B cells [5].

Due to the small amounts of antigens that can be expressed in vivo by DNA vaccines, another vaccine modality is needed to provide enough quality of the same antigens to boost and enlarge antigen-specific B cell population to produce the final high level antibody responses. At the same time, it is well known that recombinant protein vaccines or inactivated vaccines, due to their nature as the exogenous antigens, are not effective to elicit high level CD4 helper T cells, which is important for the development of antigen-specific B cells. Therefore the combination of DNA prime and the boost with either a protein vaccine or inactivated vaccine provided the complementary benefits to each other.

An adjuvant is traditionally included as part of recombinant protein vaccine formulation to enhance immunity to vaccine, presumably by engaging components of the innate immune system. If DNA priming is able to elicit high level antigen-specific B cell responses, it is logical to ask if an adjuvant is still needed for recombinant protein vaccines. The second finding from the current study is that the adjuvant component routinely included in the protein vaccine formulation may be less critical as part of the DNA prime-protein boost approach. Since antigen-specific B cells are already activated by DNA prime, the need for adjuvant to achieve the same objective is greatly reduced. This finding needs to be tested in human studies and with additional adjuvants in the DNA prime-protein boost approach to confirm that the same finding can be applied to other antigens and adjuvants. If proven feasible, the removal of adjuvant will have a profound impact to the current DNA prime-protein boost approach as it will greatly reduce the complexity and the total cost of such vaccine strategies, simplify the GMP manufacturing process and formulation, and improve vaccines’ safety as adjuvants to induce certain levels of reactogenicity as potential side effects.

The third important finding from the current study is that the intervals between prime and boost can affect the final outcome of antigen-specific antibody responses. As previously reported by NIH’s Vaccine Research Center, the maximum antibody responses were induced only after several months’ resting between the prime of a DNA vaccine expressing the HA antigen from an H5N1 virus and the boost by an inactivated H5N1 virus vaccine with matching H5 HA antigen [30,31]. The current mouse study found that an immunization schedule of Weeks 0, 4 and 8 elicited the highest gp120 antibody responses while the longer or shorter intervals were suboptimal. It is possible that the host immune system may affect the optimal resting interval between the prime and boost vaccinations. Further studies are needed, especially in humans, to find the optimal interval among prime and boost for HIV vaccines. The optimal resting intervals may also vary for other non-HIV vaccines as different antigens may require different optimal resting internals.

In summary, the finding that DNA prime and protein boost is effective in eliciting antigen-specific B cell responses further confirmed the value this approach in the development of novel vaccines based on subunit antigens, either natural or modified. While recombinant protein vaccines have been developed for the past 50 years, by combining them with a DNA vaccine component in the overall clinical product development, broader applications should be expected as we have demonstrated the utility of DNA and protein combination approach against a wide range of viral, bacterial, and parasite pathogen targets [1–3,5,34–37].
